# Molecular Characterization of Coccidia Associated with an Epizootic in Green Sea Turtles (*Chelonia mydas*) in South East Queensland, Australia

**DOI:** 10.1371/journal.pone.0149962

**Published:** 2016-02-22

**Authors:** Phoebe A. Chapman, Helen Owen, Mark Flint, Rebecca J. Traub, Thomas H. Cribb, Paul C. Mills

**Affiliations:** 1 Veterinary-Marine Animal Research Teaching and Investigation Unit, School of Veterinary Science, University of Queensland, Gatton, Queensland, Australia; 2 School of Forest Resources and Conservation, University of Florida, The Florida Aquarium’s Center for Conservation, Apollo Beach, Florida, United States of America; 3 Faculty of Veterinary and Agricultural Sciences, University of Melbourne, Parkville, Victoria, Australia; 4 School of Biological Science, University of Queensland, St Lucia, Queensland, Australia; Sonoma State University, UNITED STATES

## Abstract

In the spring of 2014, mass mortalities among wild green sea turtles occurred off the coast of south-east Queensland, Australia. The suspected causative agent was *Caryospora cheloniae*, an eimeriid coccidian implicated in previous epizootics. Necropsies were undertaken on a subset of 11 dead turtles, with subsequent histopathology and molecular analyses. All turtles returned positive PCR results for coccidial infection in various tissues; these included the brain, gastrointestinal tract, lung, kidney and thyroid. Granulomatous encephalitis was consistently observed, as well as enteritis and, less frequently, thyroiditis and nephritis. Sequencing and phylogenetic analyses indicated the presence of two distinct coccidian genotypes, presumably separate species—one associated with the brain, gastrointestinal tract and lung, and the second with the thyroid and kidney. Maximum likelihood and Bayesian inference analyses placed the first genotype closest to the lankesterellid genus *Schellackia*, rather than in the Eimeriidae, while the second was paraphyletic to the eimeriids. Presence of coccidial stages in extra-intestinal tissues of the primary host raises questions about the potential presence of intermediate or paratenic hosts within the life cycles, as well as their current placement relative to the genus *Caryospora*. This study represents the first genetic characterization of this emerging disease agent in green sea turtles, an endangered species, and has relevance for life-cycle elucidation and future development of diagnostics.

## Introduction

Green sea turtles (*Chelonia mydas*) are inhabitants of tropical and temperate waters worldwide, and are considered to be useful sentinels of ecosystem health [1]. Anthropogenic pressures have led to the species being listed as Endangered on the IUCN Red List, and Vulnerable under Australia’s *Environment Protection and Biodiversity Conservation Act* (1999). Mortality of green sea turtles due to coccidiosis has been infrequently reported, and was initially identified in mariculture environments [2, 3]. Within the waters of south east Queensland, Australia, deaths of wild green sea turtles as a result of coccidial infection generally occur with low prevalence [4]. However, an epizootic event resulting in mass mortalities was reported in the spring of 1991, where in excess of 70 wild green sea turtles were reported dead over a 6 week period [5]. Since then, infections have been reported at low frequencies [4] until the spring of 2014, when another epizootic occurred. Little is currently known about the triggers and epidemiology of these events, or the causative organism, suspected to be *Caryospora cheloniae* [5].

Infections of *C*. *cheloniae* have been reported only from marine turtles [6]. It was first reported from hatchling and juvenile turtles in a mariculture facility in Grand Cayman [2, 3], where it caused emaciation, lethargy and intestinal impaction leading to death and subsequent economic loss. The same parasite was thought responsible for the Queensland event of 1991, where affected turtles (largely sub-adults) showed clinical signs including weakness, depression and neurological disturbance, with diagnosis supported histopathologically [5]. Both previous outbreaks were diagnosed using morphological features of oocysts alone, which may fail to recognize closely related but distinct species and relies on active shedding in the feces at the time of sampling. Molecular techniques have the capacity to differentiate between morphologically similar species at any stage of development, and facilitate accurate identification [7]. Here, we use molecular methods to investigate coccidial infections at post-mortem from the recent 2014 epizootic, and report on associated pathology in various organ systems. This represents the first molecular and phylogenetic characterization of the causative organism of this emerging disease in a species of marine turtle, providing a starting point for future definitive identification, diversity studies, and outbreak comparisons.

## Materials and Methods

### Necropsy and pathology

11 dead green sea turtles presented to wildlife rehabilitation facilities, Australia Zoo Wildlife Hospital (Beerwah) and Underwater World Sea Life Aquarium (Mooloolaba), underwent post-mortem examination in September and October 2014, with an additional animal examined in January 2015. Necropsies were undertaken shortly after death/euthanasia and were completed according to standard procedure [8]. Tissue samples collected were fixed in 10% neutral buffered formalin prior to histopathological examination. Sections were cut at 5 μm and stained with haematoxylin and eosin. Further samples were stored in 70% ethanol at 4°C for molecular analysis. Samples were submitted for histopathology from all but one animal, which had been frozen prior to necropsy. In this case, samples were collected only for molecular analysis. Although necropsies were undertaken as part of a molecular/pathological investigation not requiring fecal examination, fecal samples were collected from 1 individual, with flotation performed using saturated salt solution. Gut scrapings were taken from the same turtle and examined under light microscope.

### Ethics statement

All turtles involved in this study were euthanized on humane grounds after veterinary evaluation at rehabilitation facilities, or died shortly after stranding. Euthanasia was performed by veterinarians using intravenous injection of pentobarbital. While no turtles were euthanized specifically for the purposes of this research, all work was completed under approval no. SVS/186/14/ARC issued by the University of Queensland Animal Ethics Committee. Queensland State Government approval to undertake marine research activities was granted through Marine Parks permit no. QS2014/CVL1414 and Scientific Purposes permit no. WISP09021911.

### Molecular analysis

Molecular techniques were adapted from those outlined by Chapman et al. [9]. Ethanol preserved tissues were utilized for DNA extraction using the DNeasy Blood and Tissue kit (Qiagen, Chadstone, AU). Procedure was as per manufacturer’s instructions, however the final stage was amended to use 100 μL of buffer AE (cf. 200 μL) to provide a more concentrated product.

A generic eukaryote forward primer (3F: 5′ GTT CGA TTC CGG AGA GGG A) and an apicomplexan-specific reverse primer (Api1R: 5′ TAA TCT ATC CCC ATC ACG ATG C–3′)[10] were used to amplify the *18S* region of coccidians present in tissues. PCR reactions were run at a total volume of 25 μL, comprised of 2.5 μL 10 x PCR buffer, 4 μL dNTP mix (Qiagen, Chadstone, AU) at a final concentration of 1.25 mM each, 2.5 μL of each primer at a concentration of 10 mM, 1.25 units HotStar Taq (Qiagen, Chadstone, AU) and 1 μL template DNA with the remainder made up of nuclease free water. Cycling conditions comprised an initial activation step of 94°C for 5 minutes, followed by 40 cycles of 94°C for 30 seconds, 57°C for 30 seconds and 72°C for 2 minutes, with a final extension step of 72°C for 10 minutes. Products were visualized using a 1% agarose gel stained using SYBR Safe (Life Technologies Pty Ltd, Grand Island, New York, USA) and submitted to the Animal Genetics Laboratory (School of Veterinary Sciences, University of Queensland) for purification and bidirectional sequencing.

Chromatograms were examined using Finch TV v1.4.0 [11] and aligned using the ClustalW accessory within BioEdit v7.0.9.0 [12]. A Maximum Likelihood tree was constructed using PhyML 3.0 [13] with 1000 bootstrap replicates. The TIM3 + G + I substitution model was specified based the results of a jModelTest 2.0 [14, 15] analysis of the alignment. MrBayes 3.2.4 [16, 17] was used to construct a Bayesian inference tree. As TIM3 is not supported by MrBayes, the GTR + G + I model was specified as the closest over-parameterized model [18]. One million generations were run and samples taken every 100 generations. The first 10% were discarded as burn-in, with convergence verified using Tracer v1.6 [19].

## Results

### Demographics

All 11 green sea turtles necropsied were confirmed to have coccidial infections. The animals were from a range of age and size classes (Table 1). The majority (n = 8) of these turtles were stranded in an area between Beachmere (27°7’43, 153°3’06) and Bribie Island (27°4’32, 153°9’49). Of the remaining three, two were found to the north of this area on the Sunshine Coast (Golden (26°48’55, 153°7’59) and Teewah (26°16’37, 153°3’59) beaches) with the final turtle to the south at Sandgate (27°18’43, 153°4’10). Only two turtles were classed as small immature (juveniles), with the majority being classed as large immature (sub-adults) or mature (adults). More female turtles (8) were presented than male (3).

**Table 1:** Sex, age class, locality and morphometric data for turtles with confirmed coccidiosis.

Turtle ID	Sex	CCL	Weight	Condition	Age Class	Stranding locality	PCR
AZ55579	F	82.0	n/a	Fair/good	Sub-adult	Woorim Beach, Bribie Island	Br*, St*, Lu*
AZ55627	F	94.5	52.0	Poor	Adult	Golden Beach, Caloundra	Br*, GIT, Li, Lu, Pa, Thy*, Sp^, Ki^, He
AZ55712	F	72.0	n/a	Poor	Sub-adult	Beachmere	Br*
AZ55763	F	105.0	90.0	Good	Adult	Sandgate Beach	Br*, SI*, Ki, Sp, Li, He, Lu, Thy
AZ55836	F	82.0	n/a	Good	Sub-adult	Beachmere	Br*
AZ55888	F	45.5	n/a	Poor	Juvenile	Red Beach, Bribie Island	Br*
AZ56016	F	65.0	29.0	Fair/poor	Sub-adult	Sandstone Point	Br*
AZ56054	M	79.0	n/a	Poor	Sub-adult	Beachmere	Br*
AZ56124	M	71.3	n/a	Good	Sub-adult	Bribie Passage	Br, St, Sp, Ki, He, Li, SI^, Thy, Pa, Lu
AZ56143	F	52.7	13.8	Poor	Juvenile	Godwin Beach	Br*
AZ56168	M	92.0	72.0	Fair/poor	Adult	Teewah Beach	Br*, Ki*

Abbreviations: F = female, M = male, CCL = curved carapace length, Br = brain, St = stomach, Lu = lung, Li = liver, Pa = pancreas, Thy = thyroid, Sp = spleen, Ki = kidney, He = heart, SI = small intestine, GIT = gastrointestinal tract.

* denotes positive PCR result from which a sequence was obtained.

^ denotes positive PCR result with no sequence. CCL is given in centimeters while weight is given in kilograms.

### PCR and molecular characterization

Positive PCR results for coccidia were obtained from various tissues from all 11 turtles. In each case, brain tissues were tested along with other tissues where available (Table 1). Fifteen sequences were collected from 10 of the 11 confirmed infected turtles (Table 1). Ten sequences were obtained from brain tissue, and one each from stomach, small intestine, lung, kidney and thyroid tissue. Additional positive results were obtained from a kidney, spleen and small intestine sample, however attempts at sequencing were not successful in these cases. Two distinct genotypes were identified. The first and most common genotype was obtained from all positive brain and gastrointestinal samples, as well as the lung sample. The kidney and thyroid sequences related to a second genotype. In both turtles in which this second genotype was identified, the first genotype was present in the brain. The two genotypes varied by 34/920 examined bases (96.3% similarity). Within Genotype 1, sequences varied by a maximum of four bases, whereas the two examples of Genotype 2 were identical apart from a single ambiguous base in one sequence.

**Fig 1:**
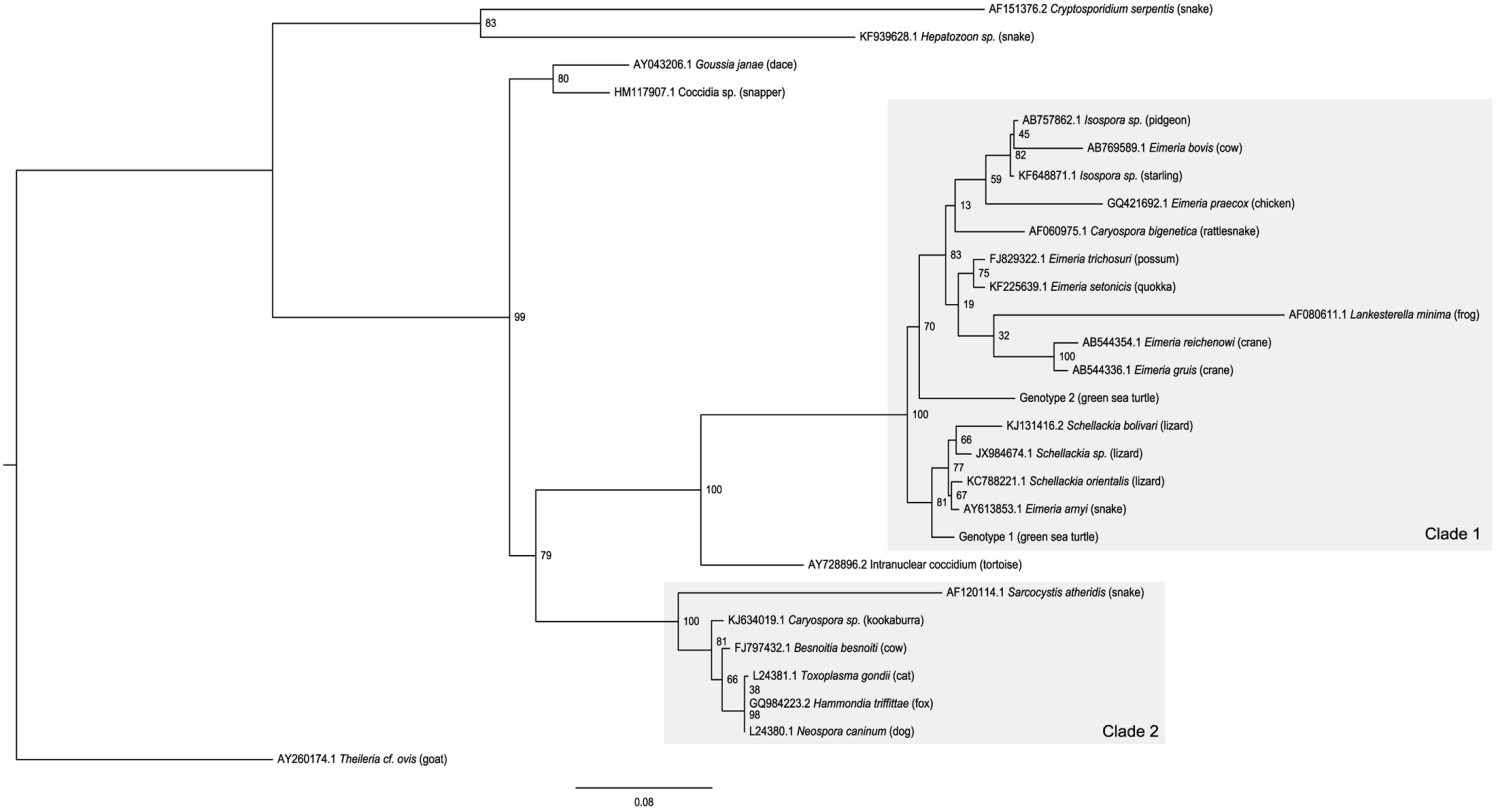
Maximum likelihood analysis of 18S partial sequences from coccidia of green sea turtles relative to a range of eimeriid, lankesterellid and cyst-forming coccidians. Numbers at each node represent bootstrap support values expressed as a percentage rounded to the nearest whole number. Scale-bar indicates the number of nucleotide substitutions per site. Species names are preceded by Genbank accession numbers, and definitive hosts follow in parentheses. Clade 1 indicates the clade containing coccidians from Eimeriidae and Lankesterellidae, while Clade 2 contains the families Sarcocystidae, Barrouxiidae and Calyptosporiidae.

**Fig 2:**
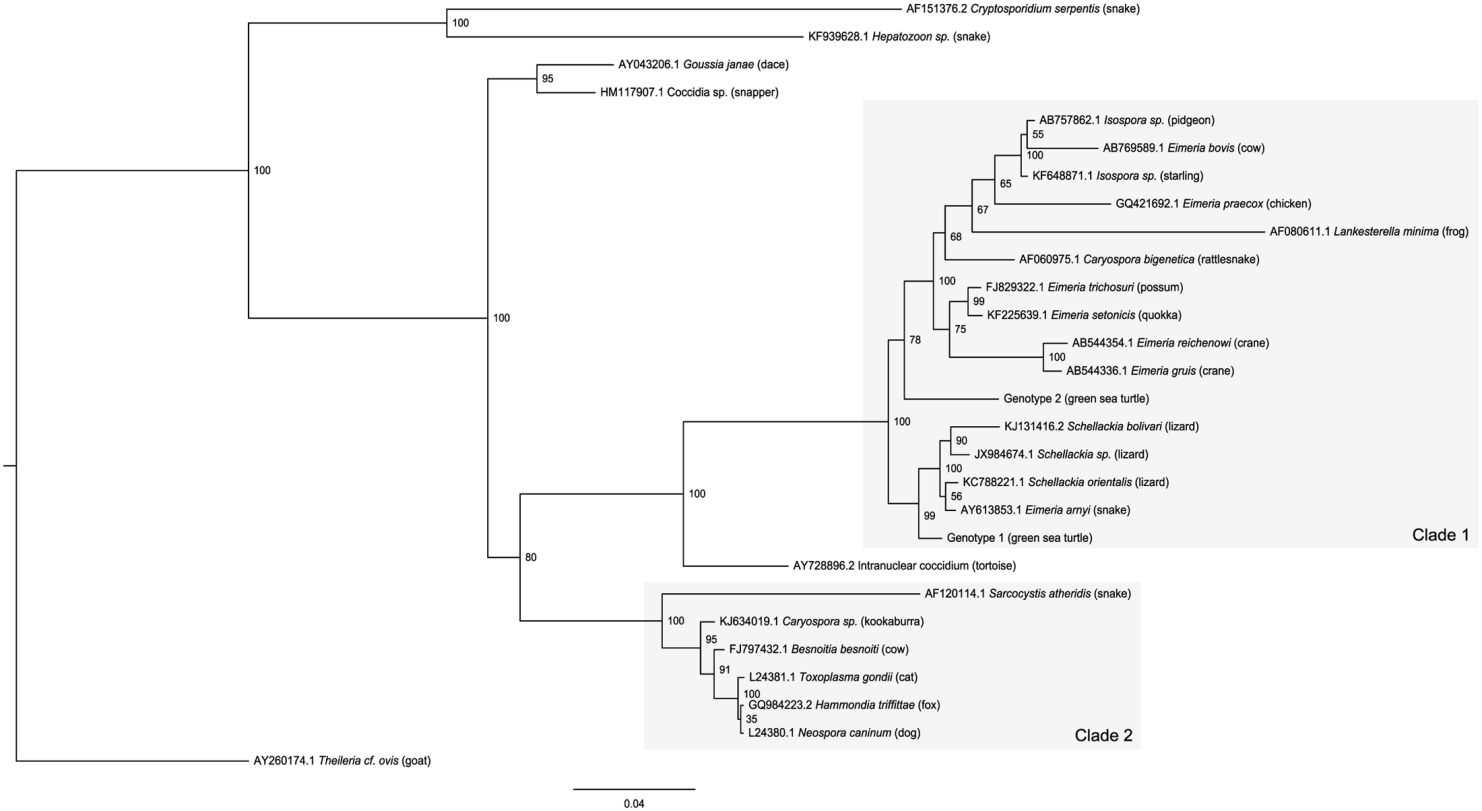
Bayesian inference analysis of 18S partial sequences from coccidia of green sea turtles relative to a range of eimeriid, lankesterellid and cyst-forming coccidians. Numbers at each node represent posterior probability values as a percentage rounded to the nearest whole number. Scale-bar indicates the number of nucleotide substitutions per site. Species names are preceded by Genbank accession numbers, and definitive hosts follow in parentheses. Clade 1 indicates the clade containing coccidians from Eimeriidae and Lankesterellidae, while Clade 2 contains the families Sarcocystidae, Barrouxiidae and Calyptosporiidae.

A BLASTN search indicated that the closest match to Genotype 1 was *Schellackia orientalis* (Genbank accession KC788221.1, 98.7% similarity), a coccidian infecting Asian lizards [20]. Genotype 2 most closely matched two species of *Eimeria* from Australian marsupials—*E*. *setonicis* [21] (quokka—KF225639.1, 96.25% similarity) and *E*. *trichosuri* [22, 23] (possum—FJ829322.1/FJ829320.1, 96.25% similarity). The maximum likelihood (Fig 1) and Bayesian inference (Fig 2) trees showed similar topology. Two major clades were formed; the first was comprised of species from the Eimeriidae and Lankesterellidae, while the second contained the cyst forming coccidia from the families Sarcocystidae, Barrouxiidae and Calyptosporiidae. Both green turtle genotypes fell in the first clade, which itself is comprised of two major clades. The *Schellackia* and green sea turtle coccidian Genotype 1 form one clade, while the eimeriid coccidians along with *Lankesterella minima* (Lankesterellidae) and green sea turtle Genotype 2 formed the second clade. Both analyses indicated that Genotype 1 was more similar to *Schellackia spp*. than to Genotype 2, however *Schellackia* species were more closely related to each other than to either green sea turtle genotype, suggesting that the latter may form their own related genus or genera. Representative sequences were submitted to Genbank (Genotype 1 accession number KT361639; Genotype 2—KT361640).

### Clinical Presentation and Gross Pathology

Turtles presented for necropsy had been examined by the receiving veterinarian at the relevant hospital prior to death or euthanasia, and were generally reported to be lethargic, minimally responsive and weak. Several were unable to dive, and at least half displayed neurological disturbance; e.g. circling, head tilt and inability to maintain level orientation in water. Rehabilitation facilities did not report diarrheic feces, however turtles were often only in care for a short period before death or euthanasia.

On necropsy, blood vessels associated with the gastrointestinal tract and adjacent mesentery were commonly engorged. In one case, parts of the intestinal mucosa showed a red discoloration. Gut fill appeared normal in the majority of cases; but 3 turtles had tightly packed digesta, predominantly in the crop/stomach. No other abnormalities were observed.

The examination of a fecal sample and gut scrapings from one individual (AZ56124) failed to yield any oocysts.

### Histopathology

**Fig 3:**
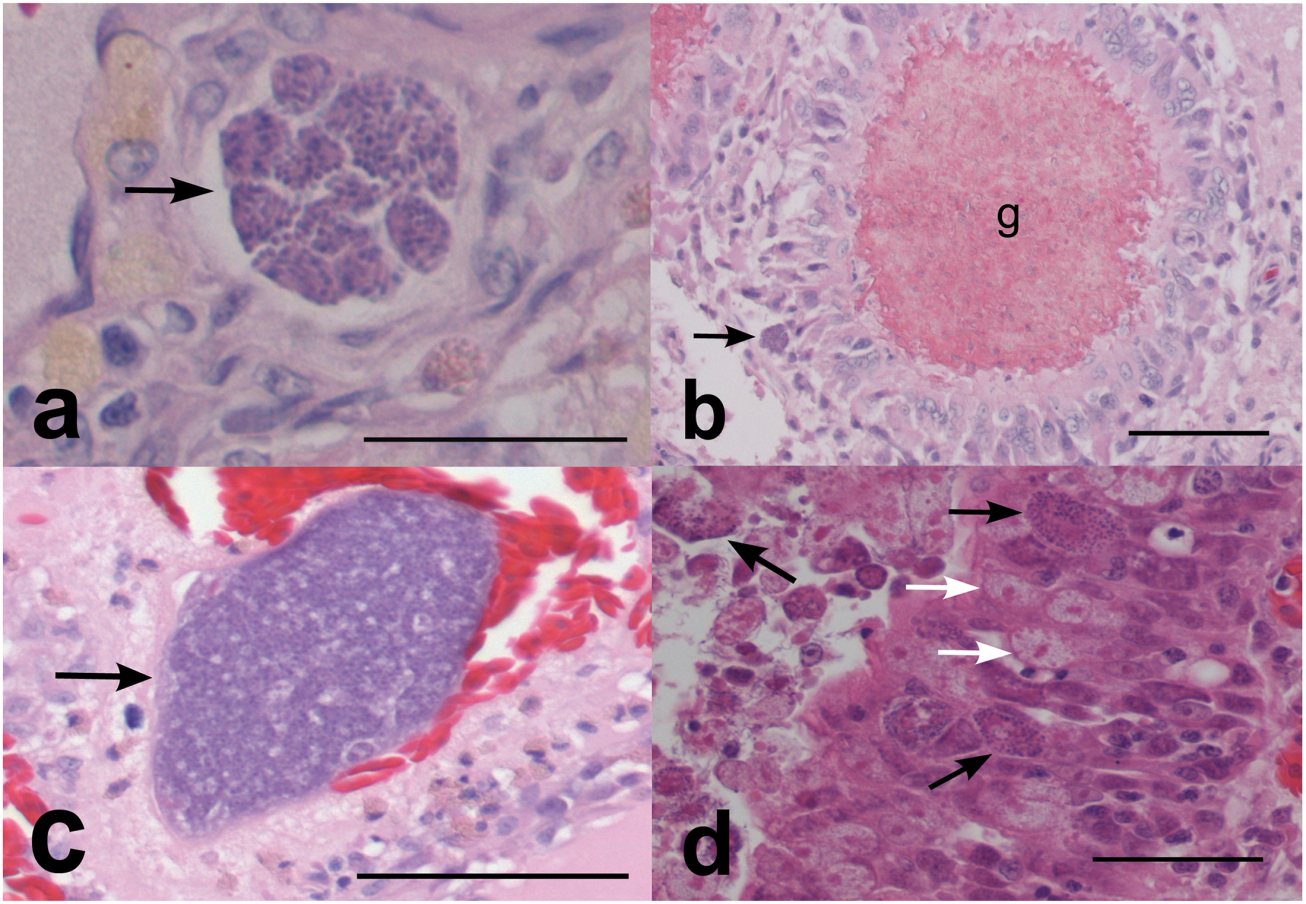
Histological views of green sea turtle coccidia. a) Typical meront arrangement (arrow) in the brain of a green turtle (56168) HE, bar = 65μ; b) granuloma (g) associated with meronts (arrow) in the brain (56168). HE, bar = 260μ; c) Aggregation of merozites (arrow) in the thyroid (56168). HE, bar = 175μ; d) meronts (black arrows) and macrogametes (white arrows) within the intestinal epithelium (56143). HE, bar = 65μ. Abbreviations: HE = Haematoxylin and eosin

Tissues from 10 turtles returning positive PCR results for apicomplexan infection were submitted for histopathologic examination. In six of these (AZ56168, AZ56143, AZ56016, AZ55888, AZ55763, AZ55712) basophilic protozoal organisms (merozoites ≤1 μm or meronts 30–80 μm–Fig 3a) were visible in the brain with moderate to severe associated granulomatous encephalitis. Encephalitis was evident in two further individuals (AZ56124, AZ55836) however the causative agent was not readily visible. Typically, multifocal pyogranulomas measuring approximately 80 μm and with necrotic centers were present (Fig 3b), although in one turtle (AZ56143) these were considerably larger (up to 500 μm). In each case, the neuropil contained populations of gitter cells, which varied from scattered to locally dense. Perivascular cuffing of vessels within the meninges or neuropil was observed in two individuals (AZ56168, AZ56016) with moderate populations of lymphocytes. Spongiosis (oedema) within the neuropil was evident in AZ55712 and AZ55888, with occasional degenerate and swollen axons (spheroids) present in affected white matter.

One individual (AZ56168) with severe meningoencephalitis also showed inflammation of the kidneys and thyroid, with associated basophilic protist stages. Interstitial fibrosis and lymphoplasmacytic inflammation were evident in both kidneys, although protists were only discernable in one. Although the identity of these protist stages could not be determined visually, PCR confirmed the presence of coccidia (Genotype 2). Within the thyroid, a multifocal to locally extensive interstitial population of lymphocytes, macrophages and likely heterophils was evident, with a nearby aggregate of protist stages (Fig 3c); tissue was not available for PCR testing. A second turtle (AZ55888) with confirmed coccidial infection of the brain also showed notable inflammation of the pancreas, liver, thyroid and kidneys, however no protists were visible and tissue was not available for PCR testing.

Despite positive PCR results, no significant brain pathology was noted in the remaining two turtles. However, one (AZ55627) had granulomatous pneumonia associated with zoites. No protozoal elements could be found in the final individual (AZ56054), although chronic nephritis was evident.

Of nine turtles where gastrointestinal samples were examined, enteritis was observed in four (AZ56124, AZ56143, AZ56016, AZ55712) the latter two of which had visible meronts and likely gametogenous stages within cells of the epithelium and/or lamina propria of the crop or intestine (Fig 3d). Enteritis was often necrotizing and included the intestines as well as the crop (ingluvitis).

## Discussion

This is the first molecular characterization of coccidia infecting green sea turtles. Given the role of these organisms in mass mortalities in the south-east Queensland region, greater understanding of their life-cycles, epidemiology and pathogenesis is of high interest to those involved in turtle conservation. Molecular data provide valuable and sensitive tools in parasite detection and identification, and can assist in epidemiological studies. Efficient and reliable diagnosis is an essential tool in the surveillance and management of disease, and blood based molecular techniques have been successfully used for the detection and diagnosis of other coccidian infections (e.g. *Toxoplasma gondii* [24] and *Sarcocystis sp*.[25]). Information resulting from this study may be of use in the development of similar diagnostic tests for green turtle coccidia. This would be an invaluable asset in epidemiological investigation of outbreaks, as well as in monitoring of treatment regime efficacy, and for rehabilitation facility management (e.g. quarantine).

The histomorphology of coccidial stages associated with Genotype 1 closely resembles those from the 1991 QLD outbreak [5]. Gordon et al.[5] reported that oocysts examined during their study resembled those originally described for *C*. *cheloniae* [2]. *Caryospora cheloniae*, the only coccidian formally reported from green sea turtles to date, is currently classified within the family Eimeriidae. Eimeriid coccidians are typically monoxenous parasites of the gastrointestinal tract [26–28]. Presence within extra-intestinal tissues of the primary host, while occasionally reported, is exceptional for this family [29, 30]. However, eimeriid species occurring in poikilothermic primary hosts (e.g. fish and reptiles) appear more likely to utilize extra-intestinal sites for development, and also to adopt heteroxenous life-cycles [31]. For coccidians (both eimeriid and non-eimeriid) with extra-intestinal life stages, the use of an intermediate host is common [27]. Infective stages of some species, for example *Toxoplasma gondii* and *Neospora caninum*, encyst in various tissues of intermediate hosts, and are passed on to a definitive host upon predation. In others (e.g. lankesterellids), infective sporozoites invade leukocytes or erythrocytes, and are subsequently ingested by haemophagous ectoparasite intermediate hosts and are subsequently transferred to new hosts. In some cases, including a number of species of *Caryospora*, heteroxenous life-cycles are facultative [32, 33]. The primary host may be infected through the traditional eimeriid route of ingestion of oocysts from the environment, or alternatively through ingestion of an infected intermediate (or paratenic) host. In previously reported cases of *C*. *cheloniae* infection, oocysts were excreted via the traditional fecal route [3, 5]. The presence of the extra-intestinal stages seen here may suggest a more complex multi-faceted life-cycle. Infection of *Caryospora* species within extra-intestinal tissues generally occurs in the intermediate host, rather than the definitive host [28, 33]. The coccidians implicated in these green sea turtle cases therefore do not appear to show a typical eimeriid life history; whether they are life-cycle dead-ends or lead to further transmission remains to be determined. Extra-intestinal phases in the definitive host (turtle) account for the unique neurological signs observed and may be indicative of adaptation to the aquatic environment and use of an intermediate/paratenic host [31]. Given that wild sea turtles are wide ranging and do not generally form dense, highly interactive populations, use of an intermediate host may facilitate the observed sudden spikes in infection numbers.

Phylogenetic analyses suggest that, of the taxa for which sequences are available, at least one of the green sea turtle coccidia (Genotype 1) is most closely related to species of *Schellackia* (Lankesterellidae) as opposed to the Eimeriidae. While unexpected, such results are not unprecedented in phylogenetic investigations of coccidia. Intraerythrocytic protists found in marsupials, initially identified as species of *Hepatozoon* based on their morphology, have been demonstrated to be more closely related to the Sarcocystidae [34–36]. *Caryospora bigenetica* and *Lankesterella minima* were contained within the broader eimeriid clade, though their exact positioning varied; the relationship between these two species, and their paraphyletic positioning relative to the eimeriids, has previously been investigated by Barta et al. [32], however with only modest support. Although *L*. *minima* and *Schellackia* spp. are both members of the Lankesterellidae, there is strong bootstrap support here (100%) for *L*. *minima’s* position and subsequent polyphyly of *Schellackia*. Another *Caryospora* sequence included in the analysis (KJ634019.1– Kookaburra)[37] has been placed with the cyst forming coccidians. Hence, the so-named *Caryospora* species considered here cluster with multiple families of coccidia. The species of *Caryospora* (and the Eimeriorina as a whole) require further work to clarify their classifications and relationships. Unfortunately, to date no genetic data for the type species of *Caryospora*, *C*. *simplex* (a parasite of Eurasian vipers), is available in public databases. Ultimately, the green sea turtle coccidians may require their own genus or genera.

Pairwise distance analysis suggests that the genetic distance between the two green sea turtle coccidian genotypes is greater than observed between some species pairs from other genera, e.g. *Schellackia* and *Eimeria*. The distance observed between the two genotypes was greater than that observed between Genotype 1 and several species of *Schellackia*. Additionally, differences in tissue predilections between the two variants were evident; all brain and gastrointestinal tract sequences were associated with Genotype 1, while Genotype 2 was isolated from kidney and thyroid samples. This distinction is consistent with the interpretation that the two genotypes represent distinct species with potentially distinct life histories. This finding also demonstrates the ability of molecular data to distinguish between closely related species where morphological features allow little resolution. Further studies will be required, including detailed morphological examination, in order to properly distinguish these two putative species and their relative roles in green sea turtle mortality events. This information will be significant in the development of future diagnostic tests and treatment regimes.

The first reports of *C*. *cheloniae* outbreaks in maricultured green sea turtles from Grand Cayman Island [2, 3] made no mention of neurological impacts in their clinical accounts, but report significant gastrointestinal pathology. Besides a consistent presence of enteritis, neurological signs were reported from several affected turtles during the 1991 event in Queensland [5]. Of the cases investigated in the present study, neurological disturbance along with lethargy and weakness were the prevalent clinical signs. Coccidial meronts in the brain were commonly observed during both Queensland studies, however no histopathological examination of the brain was mentioned in Grand Cayman. Further comparative analyses, including molecular characterization, is required to establish whether mortality events occurring in the two regions are attributable to the same coccidia species, or if other factors (e.g. environmental) may be responsible for the apparent variation in pathogenic effects.

Development of coccidia in tissues of poikilotherms has been shown to be temperature dependent [31]. The two major coccidial epizootics in Moreton Bay both occurred in the spring months. With water temperatures rising at this time after low winter temperatures, it is possible that atypical fluctuations in water warming patterns may influence coccidial development and replication rates. Similarly, seasonal factors may favor population booms or increased activity in potential invertebrate intermediate hosts. Other biotic (e.g. algal assemblages) and abiotic (e.g. water quality, flow regimes, currents) factors are also influenced by season and may affect parasite-host dynamics.

The proportion of female turtles in Moreton Bay (Moreton Banks) has previously been reported at 66% [38]. Of the affected turtles in this study, 72% were female, which despite the relatively small sample number is reflective of the reported Moreton Bay proportion. The turtle population at Moreton Banks is reported to be dominated by juvenile size classes; 89% of females and 96% of males were juvenile [38]. In this study, 75% of females were immature (juvenile or sub-adult), and 66% of males. This suggests that adult turtles were more susceptible to coccidia infection, in conflict with the results of Gordon et al. [5] who found that sub-adult and pubescent turtles were most commonly presented. However, our small sample sizes make interpretation of these patterns difficult, particularly given the narrow time-frame involved and natural shifts in population structure that occur over the year.

Coccidiosis in green sea turtles remains an understudied disease that has a significant impact on populations in southern Queensland. The data presented here demonstrates that there is much to learn about the causative organisms, as well as the taxonomy and phylogeny of the coccidia in general. Further development of tools and exploration of molecular data in conjunction with pathological and epidemiological studies will result in improved capacity to understand and manage future outbreaks.
